# Progression of Alzheimer's disease parallels unusual structural plasticity of human dentate granule cells

**DOI:** 10.1186/s40478-022-01431-7

**Published:** 2022-08-29

**Authors:** B. Márquez-Valadez, A. Rábano, M. Llorens-Martín

**Affiliations:** 1 Department of Molecular Neuropathology, Centro de Biología Molecular “Severo Ochoa” (CBMSO), Spanish Research Council (CSIC), Universidad Autónoma de Madrid (UAM) (Campus de Cantoblanco), c/Nicolás Cabrera 1, 28049 Madrid, Spain; 2 Center for Networked Biomedical Research On Neurodegenerative Diseases (CIBERNED), Madrid, Spain; 3 Neuropathology Department, CIEN Foundation, Madrid, Spain

**Keywords:** Alzheimer's disease, Hippocampus, Morphological alterations, Dentate granule cells, Dentate gyrus, Golgi-Cox staining

## Abstract

Alzheimer´s disease (AD), the most common form of dementia in industrialized countries, severely targets the hippocampal formation in humans and mouse models of this condition. The adult hippocampus hosts the continuous addition of new dentate granule cells (DGCs) in numerous mammalian species, including humans. Although the morphology and positioning of DGCs within the granule cell layer (GCL) match their developmental origin in rodents, a similar correlation has not been reported in humans to date. Our data reveal that DGCs located in inner portions of the human GCL show shorter and less complex dendrites than those found in outer portions of this layer, which are presumably generated developmentally. Moreover, in AD patients, DGCs show early morphological alterations that are further aggravated as the disease progresses. An aberrantly increased number of DGCs with several primary apical dendrites is the first morphological change detected in patients at Braak-Tau I/II stages. This alteration persists throughout AD progression and leads to generalized dendritic atrophy at late stages of the disease. Our data reveal the distinct vulnerability of several morphological characteristics of DGCs located in the inner and outer portions of the GCL to AD and support the notion that the malfunction of the hippocampus is related to cognitive impairments in patients with AD.

**Supplementary Information:**

The online version contains supplementary material available at 10.1186/s40478-022-01431-7.

## Introduction

Alzheimer's disease (AD) is the most common form of dementia. Its increasing prevalence is exacerbating the healthcare burden worldwide. From a histopathological perspective, this disease is characterized by extracellular deposits of amyloid-β (Aβ), and intracellular neurofibrillary tangles formed by hyperphosphorylated tau. The accumulation of the latter protein is used to classify AD severity on the basis of the progressive spread of tangles across distinct regions of the brain [1]. AD patients typically exhibit memory impairments, progressive cognitive dysfunction, and changes in mood and behavior [2].

The hippocampus is one of the brain areas most affected in patients with AD [1]. This region is divided into the CA1, CA2, CA3/4, and dentate gyrus (DG) subfields. The perforant pathway, which is formed by the axons of pyramidal neurons in the entorhinal cortex (EC), is one of the main cortical inputs that reach the DG. The aforementioned axons establish the first synapse with the dendrites of dentate granule cells (DGCs). The morphological maturation of this cell population is crucial for the establishment of appropriate afferent connections from the perforant pathway. DGC axons (named Mossy fibers) contact the CA3 and CA2 hippocampal regions [3–5]. The third synapse of the circuit is established between Schaffer collaterals (the axons of the CA3 pyramidal neurons) and dendrites of CA1 pyramidal neurons, which project back to the subiculum and the EC. The classic hippocampal tri-synaptic circuit (EC-DG-CA3-CA1) is crucial in learning and memory [6].

The hippocampus is one of the few regions of the brain to host adult neurogenesis, a phenomenon described in numerous mammalian species, including humans [7–9]. As a result of adult hippocampal neurogenesis (AHN), new DGCs are incorporated into the hippocampal circuit throughout life. These cells are the most abundant neuronal population of the DG and their morphology is closely related to their function and connectivity. DGCs have a primary apical dendrite that emerges from the soma and is vertically oriented towards the molecular layer (ML) [10–12]. Primary apical dendrites remain poorly branched until they reach the ML, where they become extensively branched and receive excitatory synaptic inputs from the medial and lateral EC. The classical morphology of DGCs described above has been named the “Y-shape” [13]. In rodents, both the dendritic morphology and cell position within the granule cell layer (GCL) vary according to the developmental origin of DGCs [12, 14, 15]. In this regard, developmentally generated DGCs are preferentially located in the outer GCL (in close contact with the ML) and show wider dendritic angles and more primary dendrites than adult-born DGCs. In contrast, the latter remain in the inner third of the GCL (near the hilus) and, depending on their degree of maturation, they show dendritic trees of variable complexity [15]. However, whether similar morphological features are present in human DGCs and the extent to which these morphologies are targeted by distinct forms of neurodegeneration remain elusive to date.

Interestingly, patients with AD [16, 17] and animal models of this disease [18] show morphological alterations in the dendritic trees and spines of CA1 and CA3 pyramidal neurons. Moreover, the DGCs of AD patients with severe dementia show a reduced density of dendritic spines and total dendritic length [11, 19, 20]. Similar alterations are reported in patients with frontotemporal dementia [21]. Remarkably, the percentage of DGCs with more than one primary apical dendrite is increased in patients with AD and mouse models of this condition [20]. This so-called “V-shape” pathological phenotype sharply contrasts with the classical “Y-shape” phenotype observed in neurologically healthy control subjects and wild-type mice [20]. Whether such an *unusual form of dendritic plasticity* [22] drives the morphological and functional alterations observed in DGCs during the progression of AD or whether such plasticity is a consequence of the disease remains to be elucidated.

The number of immature DGCs decreases gradually throughout AD progression [9]. Moreover, patients with mild and severe cognitive impairment show fewer immature neurons [23]. In contrast, the number of mature DGCs remain stable in these patients [9]. Therefore, AHN impairments have been proposed to underlie cognitive dysfunction in AD [24]. Our data reveal that human DGCs located in the inner and outer GCL exhibit specific morphological features and differential susceptibility to neurodegeneration, which might be related to their distinct developmental origin. Moreover, the DGCs of patients with AD show early morphological alterations, which are aggravated as the disease progresses. Taken together, these results suggest that the morphological and functional impairment of DGCs parallels hippocampal malfunction in patients with AD.

## Materials and methods

### Human subjects

Twenty-two subjects (5 neurologically healthy controls and 17 patients with AD) were included in this study. Additional file 1: Figure S1 includes detailed epidemiological data for these individuals. The use of brain tissue samples was coordinated by the local brain bank (Banco de Tejidos CIEN, Madrid, Spain), following national laws and international ethical and technical guidelines on the use of human samples for biomedical research purposes [25, 26]. These guidelines included obtaining informed consent for brain tissue donation from living donors and the approval of the whole donation process by the Ethical Committee of the Banco de Tejidos CIEN (Committee approval references #15-20,130,110, # CEI PI 30_2020-v2, and # S19013). Samples were collected at the Banco de Tejidos CIEN (Madrid, Spain), the Hospital Clínico Universitario Virgen de la Arrixaca (Murcia, Spain), and the Biobanco del Hospital Universitario Reina Sofía (Córdoba, Spain). The specimens were neuropathologically classified at the Banco de Tejidos CIEN by experienced neuropathologists. To determine the Braak-Tau stage, Tau phosphorylation (AT100 epitope) in the anterior hippocampus, prefrontal, parietal, and temporal associative isocortex, and primary visual cortex was quantified following previously described protocols [1, 27].

### Human hippocampal dissection

After brain extraction, a mid-sagittal section was made to separate the right and left hemispheres. To dissect the whole hippocampus, the posterior poles of the mammillary bodies and the uncus were first identified [28]. Afterwards, a coronal 2-cm-thick slice of the whole hemisphere was obtained at this anatomical level. Anatomical references external to the hippocampus were established to select the same sampling region for all subjects and to avoid any putative anatomical bias caused by hippocampal atrophy in patients with AD. After identification of the aforementioned anatomical references, a ⁓1-cm-thick hippocampal sample corresponding to the posterior portion of the anterior hippocampus was dissected on ice and rapidly immersed in Golgi solution.

### Golgi staining

Human hippocampal samples were incubated at room temperature in Golgi-Cox staining solution A/B (FD Rapid Golgi Stain™ Kit, FD Neurotechnologies, INC.) for 14 d protected from light. After a 48-h incubation in solution C at 4 °C, hippocampal tissue blocks were included in a 10% sucrose-4% agarose solution, obtaining 150 µm-thick sections in a Leica VT1200S vibratome. These sections were then mounted on gelatin-coated glass slides and air-dried for ~ 20 min. The Golgi-Cox reaction was then performed following the manufacturer´s instructions. Sections were finally counterstained with toluidine blue to identify anatomical structures. Between 5 and 8 Golgi-stained sections per subject were analyzed.

### Morphometric analysis of human hippocampal DGCs

Following a previously described protocol, DGCs were randomly selected and traced under an inverted Axiovert200 Zeiss optical microscope (40 × dry objective) coupled to a *camera lucida* [21]. To delineate the inner and outer halves of the GCL, the thickness of this structure was measured and divided into two halves. Cells located between the aforementioned division line and the hilus were assigned to the “inner” category, whereas those located between the division line and the ML were assigned to the “outer” category (Additional file 2: Figure S2). Neurons located at the inner (near the hilus) or outer (near the ML) halves of the GCL were analyzed separately. Cells were traced using the NeuronJ plugin for Fiji software (ImageJ version 1.50e; http://rsb.info.nih.gov/ij/), and reconstructions were used to determine total dendritic length and dendritic branching (Sholl`s analysis). Sholl`s analysis was performed using the Sholl Analysis plugin for Fiji [20, 21]. Primary apical dendrites that emerged directly from the soma and ending-tips of the dendritic tree were counted manually. To evaluate the maximum dendritic span [15], the angle connecting the most external branch-tips of dendrites and the center of the soma was measured using the Angle tool for ImageJ. The number of branches of each order was calculated using the centrifugal method, which assigns order 1 to the dendrites that emerge primarily from the soma and increases the order by 1 at each branch point [15]. The neuronal soma was manually drawn, and its area was measured using Fiji [29]. The dendritic complexity index (DCI) was calculated for each neuron as previously reported [30], using the following formula:

$$DCI= \frac{\sum\; branch \;orders +\# ending tips}{\#\; of\; primary \;dendrites}\times total \;dendritic \;length$$

### Acquisition of representative images

Images were acquired under a THUNDER Imager Tissue microscope, equipped with a Leica DFC9000 GTC VSC-09991 camera (Leica Microsystems Ltd., Wetzlar, Germany). For Extended Depth of Field (EDF) images containing the whole hippocampus, an HC PL APO 20x/0.80 DRY objective and Tile Scan acquisition mode were used. For representative images of neuronal morphology, an HC PL APO 40x/0.95 and a 63x/0.95 oil objective lens were used. Images were processed using the Leica Application Suite X (LAS X) software provided by the manufacturer (Leica Microsystems Ltd., Wetzlar, Germany).

### Statistical analysis

Statistical analyses were performed using GraphPad Prism 9 or SPSS v.17 software. The Kolmogorov–Smirnov normality test was used to check the normality of sample distribution. Comparisons between two experimental groups were performed using an unpaired two-sided Student`s *t*-test in the case of normal sample distribution or a Mann–Whitney *U* test when normality could not be assumed. To compare more than two experimental groups, data were analyzed by either one-way or repeated-measures ANOVA test with Tukey`s post-hoc comparisons in cases of normal simple distribution, or by a Kruskal–Wallis test with Dunn`s post-hoc analyses when normality distribution could not be assumed. Two-way ANOVA and mixed-effect model analysis were used to assess the effects of more than one variable. For comparison of qualitative variables, a Pearson *X*^2^-test was applied. A mixed-effect model analysis (SPSS v.17, Factors: Diagnose, position, age, subject; covariate: cells. Fixed effects: Diagnose and cell positioning; Random effects: age and subject) was performed to determine the putative effects of age and inter-individual variation on the morphological parameters studied. A two-sided Pearson´s correlation test was applied to assess correlations between the age of the subjects and morphometric determinations (Additional file 3: Figure S3). Graphs represent mean values ± S.E.M. A 95% confidence interval was used for statistical comparisons. The detailed results of statistical analyses are included in the Additional file 5: Extended data file.

## Results

### Morphological characterization of human DGCs in neurologically healthy control subjects

**Fig. 1:**
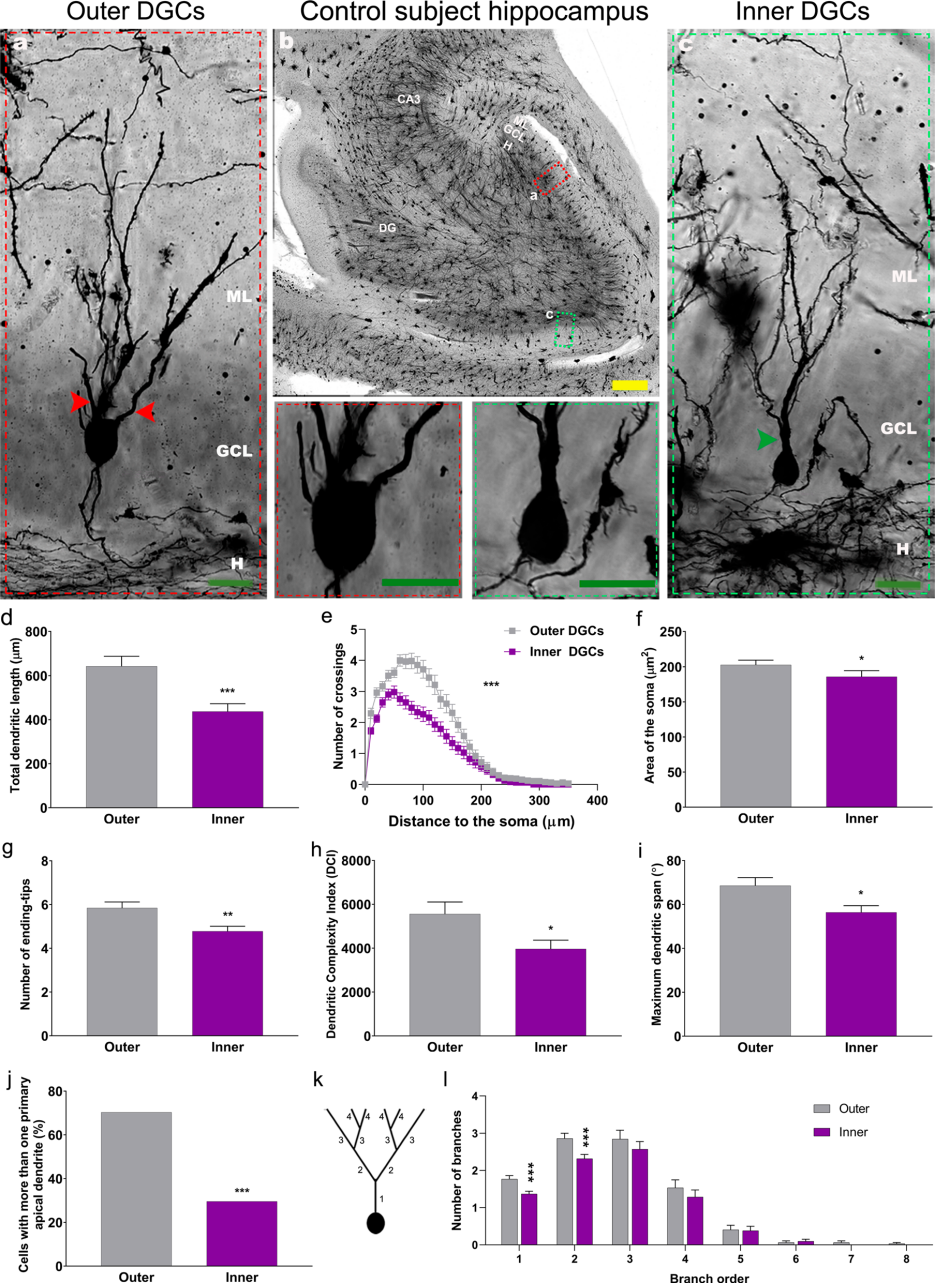
Morphological features of human dentate granule cells (DGCs) located in the outer and inner granule cell layer (GCL) of neurologically healthy control subjects. **a-c** Representative images of Golgi-stained human hippocampi and high-power magnification images showing the somata and primary dendrites of DGCs. **d** Total dendritic length. **e** Sholl´s analysis. **f** Area of the soma. **g** Number of ending-tips. **h** Dendritic complexity index (DCI). **i** Maximum dendritic span. **j** Percentage of cells with more than one apical primary dendrite. **k** Schematic representation of dendrite branch orders. **l**. Number of dendrites in each branch order. Yellow bar: 500 µm. Green bar: 20 µm. DG, dentate gyrus; GCL, granule cell layer; H, hilus; ML, molecular layer. Red and green arrowhead: apical primary dendrites. n = 127 cells obtained from 5 neurologically healthy control subjects. * 0.05 > *P* ≥ 0.01; ** 0.01 > *P* ≥ 0.001; and *** *P* < 0.001. Asterisks represent statistically significant differences in unpaired two-tailed Mann–Whitney U or Chi-squared test

Previous descriptions of the general morphological features of human DGCs are available in the literature [11, 20]. However, the extent to which the morphology of these cells varies depending on their positioning within the GCL remains poorly studied. To thoroughly characterize the morphology of DGCs located in distinct sub-regions of the GCL, morphometric determinations were performed on outer and inner DGCs separately. Compared to outer DGCs, inner DGCs exhibited shorter dendritic trees (*U*_1, 127_ = 1259, *p* < 0.001) (Fig. 1a-d) and reduced dendritic branching (Repeated measures ANOVA, Greenhouse–Geisser Interaction *F*_1, 125_ = 13.582, *p* < 0.001) (Fig. 1e). The soma area of inner DGCs was smaller than that of outer DGCs (*U*_1, 127_ = 1533, *p* = 0.020) (Fig. 1a-c, and f). Furthermore, inner DGCs presented fewer ending-tips (*U*_1, 127_ = 1435, *p* = 0.004) (Fig. 1g). The dendritic complexity (DCI index) revealed that inner DGCs presented less complex dendritic trees (*U*_1,127_ = 1477, *p* = 0.0499) (Fig. 1h), and a smaller dendritic span (*U*_1,127_ = 1500, *p* = 0.013) (Fig. 1i) than outer ones. A reduced percentage of inner DGCs with more than one primary apical dendrite was also observed (*X*^2^_1,127_ = 14,997; *p* < 0.001) (Fig. 1j). Accordingly, inner DGCs showed fewer proximal branches than outer DGCs (1st order: *U*_1,127_ = 1284, *p* < 0.001; 2nd order: *U*_1,127_ = 1357, *p* =  < 0.001) (Fig. 1k and l), despite having a similar number of distal branches. Pearson´s correlation test (Additional file 3: Figure S3 and Additional file 5: Extended data) revealed no effect of subject´s age on the morphology of DGCs in neurologically healthy control subjects.

Taken together, these results indicate that inner DGCs exhibit a less complex morphology than their outer counterparts—a finding that is compatible with a more immature neuronal phenotype [12, 15, 31].

### Morphological alterations in the DGCs of patients with AD

**Fig. 2:**
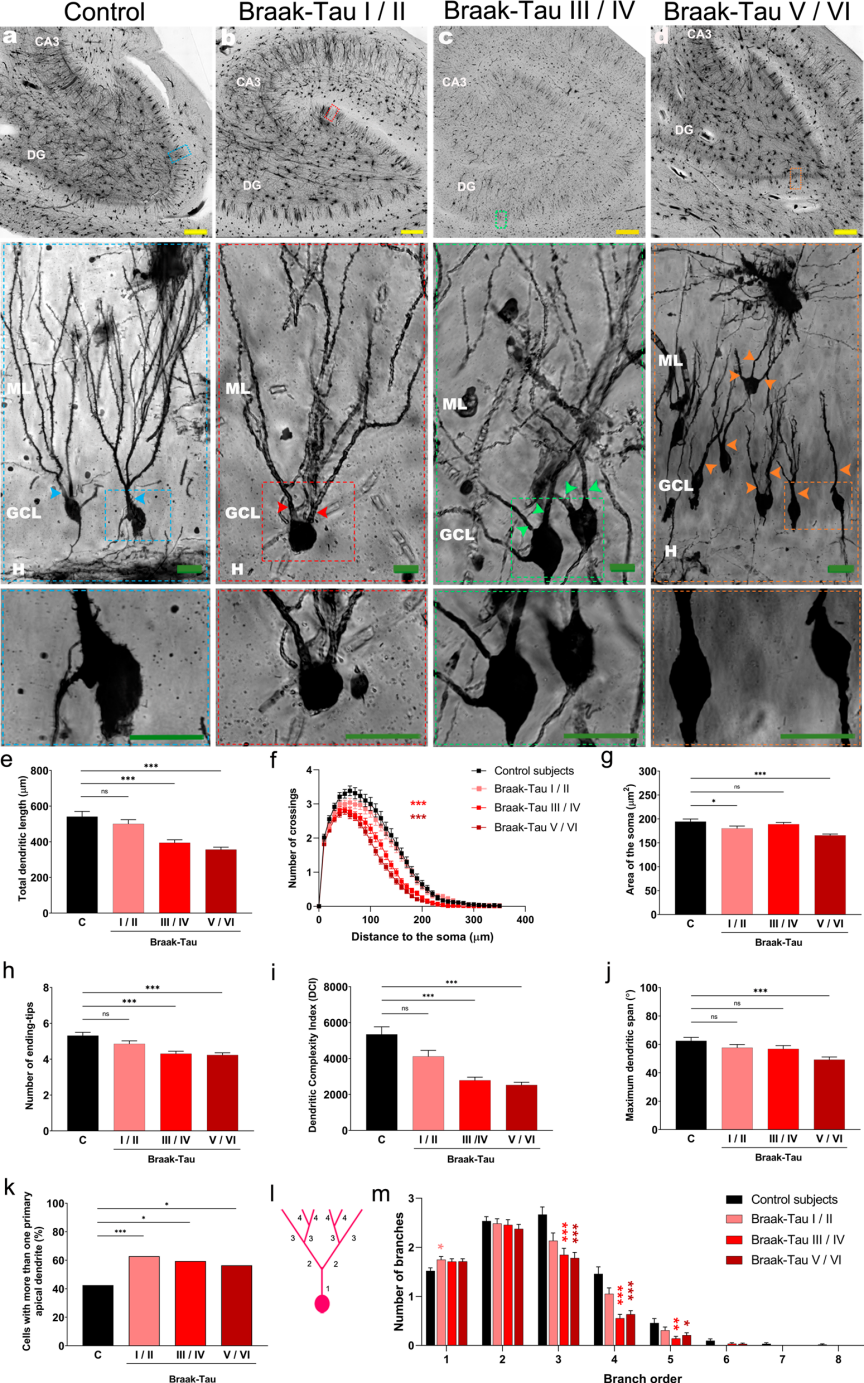
Morphological alterations of human dentate granule cells (DGCs) during the progression of Alzheimer´s disease (AD). **a-d** Representative images of Golgi-stained hippocampi and high-power magnification images showing the somata and primary dendrites of DGCs from control subjects and AD patients at distinct Braak-Tau stages. **e** Total dendritic length. **f** Sholl´s analysis. **g** Area of the soma. **h** Number of ending-tips. **i** Dendritic complexity index (DCI). **j** Maximum dendritic span. **k** Percentage of cells with more than one apical primary dendrite. **l** Schematic representation of dendrite branch orders. **m**. Number of dendrites in each branch order. Yellow bar: 500 µm. Green bar: 20 µm. DG, dentate gyrus; GCL, granule cell layer; H, hilus; ML, molecular layer. Colored arrowheads: apical primary dendrites. n = 127 cells obtained from 5 neurologically healthy control subjects and 512 cells obtained from 17 AD patients. * 0.05 > *P* ≥ 0.01; ** 0.01 > *P* ≥ 0.001; and *** *P* < 0.001. Asterisks represent statistically significant differences in Tukey`s (ANOVA) or Dunn`s (Kruskal–Wallis) post-hoc analyses, or Chi-squared test

To study the morphology of DGCs throughout AD progression, we studied patients distributed along the six neuropathological stages of the disease (Braak-Tau stages (I-VI)). For graphical representation and statistical analyses, subjects were grouped into the following categories: Control, Braak-Tau I/II, Braak-Tau III/IV, and Braak-Tau V/VI [32]. We analyzed total DGCs (Fig. 2) and outer/inner (Additional file 4: Figure S4) DGCs separately.

The mixed-effects model analysis revealed an effect of Braak-Tau stage (*F* = 8.823, *p* = 0.002) and cell positioning (*F* = 34.24, *p* < 0.001) on total dendritic length. Moreover, a statistically significant interation between Braak-Tau stage*cell positioning (*F* = 2.742, *p* = 0.042) pointed to differential vulnerability of this parameter in inner/outer DGCs to progression of the disease (Additional file 5: Extended data). In this regard, compared to control subjects, the total dendritic length of DGCs decreased progressively as Braak-Tau stages advanced (*K*_3,639_ = 43.86, *p* < 0.001), although it remained unchanged in patients at Braak-Tau I/II stages (Fig. 2a’-d’, and e). Although similar alterations were observed in outer DGCs (Additional file 5: Extended data), inner DGCs showed a decreased total dendritic length only at Braak-Tau V/VI stages (Additional file 4: Figure S4a and Additional file 5: Extended data). Sholl´s analysis revealed progressively decreased dendritic branching in AD patients at Braak-Tau III/IV stages and onwards (Repeated measures ANOVA, Greenhouse–Geisser Interaction *F*_*3,635*_ = 19.685, *p* < 0.001) (Fig. 2f). A similar decrease was observed in outer DGCs (Additional file 5: Extended data), although alterations in dendritic branching of inner DGCs were observed only in patients at Braak-Tau V/VI stages (Additional file 4: Figure S4g-h and Additional file 5: Extended data). The mixed-effects model analysis revealed an effect of Braak-Tau stage (*F* = 3.375, *p* = 0.048) on DGC soma area (Additional file 5: Extended data). In this respect, this parameter was reduced in AD patients at Braak-Tau I/II and V/VI stages (*K*_3,639_ = 37.43, *p* < 0.001) (Fig. 2a’-d’ and 2 g). Similar alterations were observed in outer DGCs (Additional file 5: Extended data), but no changes in this parameter were detected in inner DGCs.

The mixed-effects model analysis revealed an effect of Braak-Tau stage (*F* = 5.995, *p* = 0.012) and cell positioning (*F* = 38.931, *p* < 0.001) on the number of ending-tips (Additional file 5: Extended data). Post-hoc analyses revealed that this parameter decreased progressively as the disease advanced (*K*_3,639_ = 26.16, *p* < 0.001), reaching statistical significance in patients at Braak-Tau III/IV stages and onwards (Fig. 2h). In this case, similar alterations were observed for outer and inner (Additional file 4: Figure S4c and Additional file 5: Extended data) DGCs when these cells were analyzed separately.

The mixed-effects model analysis revealed an effect of Braak-Tau stage (*F* = 11.789, *p* = 0.001) and cell positioning (*F* = 4.139, *p* = 0.042) on the DCI. DGCs showed a progressive decline in this parameter (*K*_3,639_ = 47.07, *p* < 0.001), which reached statistical significance in patients at Braak-Tau III/IV and V/VI stages (Fig. 2i). Similar reductions in the DCI were observed in outer and inner DGCs (Additional file 4: Figure S4d and Additional file 5: Extended data). Similarly, the mixed-effects model analysis revealed an effect of Braak-Tau stage (*F* = 7.217, *p* < 0.001) and cell positioning (*F* = 59.121, *p* < 0.001) on the maximum dendritic span, which was reduced in patients at Braak-Tau V/VI stages (*K*_3,639_ = 19.96, *p* < 0.001) (Fig. 2j).

The percentage of DGCs with more than one primary apical dendrite was higher in AD patients regardless of Braak-Tau stage (Pearson *X*^*2*^-test, *X*^*2*^_(3,639)_ = 12,844; *p* ≤ 0.005) (Fig. 2k and Additional file 5: Extended data), thereby indicating that this parameter is markedly altered early in disease progression. The number of proximal branches increased in patients at Braak-Tau I/II stages (1st order; *K*_3,639_ = 9.804, *p* = 0.02), whereas that of distal branches was reduced in patients at Braak-Tau III/IV and V/VI stages (Fig. 2l-m) (3rd order, *K*_3,639_ = 22.82, *p* < 0.001, 4th order, *K*_3,639_ = 37.84, *p* < 0.001; and 5th order, *K*_3,639_ = 13.69, *p* = 0.003). Similar alterations were observed in outer and inner DGCs (Additional file 4: Figure S4i-k and Additional file 5: Extended data).

To rule out the effect of any potential age-driven or inter-individual variation on the morphological changes detected, we performed Pearson´s correlation tests (Additional file 3: Figure S3) and a mixed-effects model analysis (Additional file 5: Extended data). None of the morphological parameters examined in the inner/outer/total DGCs showed statistically significant Pearson´s correlations with age (Additional file 3: Figure S3). Moreover, the mixed-effects model analyses revealed no major effect of age or inter-individual variations on any of the parameters studied (except for the maximum dendritic span, which showed limited subject and age-dependent variations). Detailed results of these analyses are included in the Additional file 5: Extended data.

Taken together, these results indicate that most of the morphological alterations exhibited by DGCs in AD patients start to be observed at Braak-Tau III/IV stages. However, two parameters, namely the reduction in the area of the soma and the presence of several primary apical dendrites, are altered in the initial stages of the disease (Braak-Tau I/II stages). Strikingly, the Braak-Tau stages in which inner and outer DGCs show specific morphological alterations differ, thereby suggesting the putative differential vulnerability of some of their morphological features to specific pathological mechanisms triggered in the AD brain.

## Discussion

The hippocampus holds the capacity to generate new DGCs throughout life. In rodents, newborn and developmentally generated DGCs show distinct morphological features and positioning within the GCL. However, similar observations have not been reported in humans to date. The axons of EC layer II pyramidal neurons are the main excitatory inputs (the perforant pathway) received by DGCs. This projection is crucial for learning and memory [6], and it is one of the first to show degeneration in patients with AD [1, 32–34]. Given that both the dendritic morphology and cell positioning of DGCs might influence their synaptic output and function, we addressed the putatively differential morphology of DGCs located in distinct positions of the GCL of the human DG, as well as the presumably differential vulnerability of inner and outer DGCs to the progression of AD.

### The morphological features of human DGCs are related to their position within the GCL

The morphology and positioning of DGCs within the GCL have been characterized in rodents, rhesus monkeys, and baboons [12, 15, 35, 36] but remain poorly understood in humans. In rodents, developmentally generated DGCs occupy outer positions of the GCL, whereas adult-born DGCs are preferentially located in the inner GCL [14, 15]. According to their differential developmental origin, inner DGCs show a lower total dendritic length than outer DGCs in rodents [12, 31] and non-human primates [36]. This observation points to the preservation of the DG developmental pattern across the entire mammalian phylogenetic scale. A previous report by our group showed that the most immature Doublecortin (DCX)^+^ human DGCs are located at the SGZ and show unpolarized horizontal neurites. As these cells start to express markers of differentiatied neurons, they progressively shift their positioning from the SGZ to the GCL and start to vertically orient their dendritic trees [9, 37]. In this study, we examined, for the first time, the morphology of human DGCs located at distinct positions of the GCL. Our results demonstrate that inner DGCs present less complex morphological features than their outer counterparts. These characteristics include shorter and less complex dendrites, as well as reduced soma area, dendritic span, and DCI (Fig. 1). These morphological features of inner DGCs are compatible with a putatively more immature phenotype, similar to that observed in immature adult-born murine DGCs [31], and with the reported occurrence of AHN in the human DG [9, 23, 38]. Although birth-dating studies would be needed to experimentally support this hypothesis, our data show that DGCs located in the inner half of the human GCL exhibit morphological features that are potentially compatible with a postnatal origin. Moreover, the distinct dendritic features of inner and outer human DGCs might reflect their differential innervation from the perforant pathway, which may have profound implications for the functioning of the entire hippocampal circuit.

In rodents, most DGCs show a single primary apical dendrite emerging from the soma [15, 31]. Conversely, primate DGCs show basal and several apical dendrites [11, 36]. Our results show that the latter features are prominent in human DGCs located in the outer GCL but mostly absent in inner DGCs (Fig. 1j). In this regard, neuronal activity inversely correlates with the number of primary apical dendrites of murine DGCs during spatial exploration [39]. Further studies are needed to determine the significance, at the circuit level, of the distinct morphological features shown by inner and outer human DGCs. Nevertheless, our results would be compatible with the existence of distinct subclasses of DGCs putatively contributing in a differential manner to specific hippocampal functions.

### Unusual structural plasticity of human DGCs is observed during AD progression

AD is characterized by progressive cognitive decline. Clinical manifestations of the disease are often preceded by several decades of prodromic neuropathological and systemic disturbances. To the best of our knowledge, our data provide the first evidence of early morphological alterations of DGCs in AD patients at Braak-Tau I/II stages, most probably before the onset of the most evident clinical symptoms. Whether these morphological changes appear early during the disease progression or are a consequence of severe neurodegeneration remains to be elucidated. Strikingly, the first morphological alterations (namely a reduction in the soma area and the appearance of two or more primary apical dendrites (Fig. 2g and k)) observed in patients at Braak-Tau I/II stages occur in the proximal region of DGCs, and, therefore, might be related to the malfunctioning of the subcellular machinery of these cells. In this regard, the accumulation of phosphorylated Tau has been reported to impact the functioning of the endoplasmic reticulum [40], mitochondria [41], Golgi apparatus [42, 43], and autophagy [44] of hippocampal neurons. Moreover, distinct non-cell-autonomous perturbations of DG homeostasis may partially drive the aberrant phenotype exhibited by DGCs in AD patients. In this respect, pro-inflammatory stimuli alter DGC morphology in mice [45] via activation of glycogen synthase kinase 3β (GSK-3β) [46]. In fact, the overexpression of this kinase triggers equivalent morphological alterations of DGCs as those observed in patients with AD [20]. Nevertheless, several primary apical dendrites are noticeable throughout all Braak-Tau stages and they also appear in mouse models of this disease [20]. Therefore, our data point to this *unusual structural plasticity* exhibited by DGCs as a novel and early neuropathological hallmark of AD.

Interestingly, some authors have suggested that the reduced incorporation of new DGCs caused by early AHN impairments in patients at Braak-Tau I stage [9] might initiate the degeneration of the EC due to the loss of axonal targets [24]. Subsequently, the massive cell death of EC layer II pyramidal neurons [33, 47] would cause axonal denervation from the perforant pathway and loss of synaptic contacts made onto DGCs. The latter phenomena might underlie the further DGC dendritic atrophy observed in the intermediate (Braak-Tau III/IV) and late (Braak-Tau V/VI) stages of the disease. Interestingly, both AHN impairments and DGCs morphological alterations might be related to the deterioration of declarative memory observed in patients with this condition, as they represent the disconnection of the hippocampus from its major afferent pathway [48]. In fact, the AHN rate has been related to cognitive scores in patients with mild and severe memory impairments [23].

Although both inner and outer DGCs exhibit a variety of morphological alterations during the progression of AD, our data reveal the differential vulnerability of individual morphological features in these two cell subpopulations. For instance, outer DGCs start to show generalized dendritic atrophy in patients at Braak-Tau III/IV stages, whereas this atrophy in inner DGCs is observed only at Braak-Tau V/VI stages. In contrast, the latter cells exhibit an early increase in the number of primary apical dendrites at Braak-Tau I/II stages, whereas this alteration starts to be observed at Braak-Tau V/VI in outer DGCs. The putatively selective vulnerability of inner and outer DGCs to AD progression might be related to the differential innervation of these cells by specific components of the hippocampal circuitry. Moreover, their variable vulnerability is compatible with a postnatal origin of inner DGCs and the proximity of these cells to the subgranular zone, a specialized environment densely populated by astrocytes, microglia, and a profuse vascular network [37]. Importantly, all these elements may either buffer or amplify the negative effects of local neuroinflammation and accumulation of Aβ, and hyperphosphorylated Tau [24] and thereby underlie the higher vulnerability of inner DGCs to the toxic environment affecting the DG in AD.

## Conclusions

Our data bring to light distinct morphological features of DGCs located in the inner and outer portions of the human DG. The morphology of inner DGCs might be compatible with the postnatal origin of these cells. Moreover, we show that morphological alterations of DGCs are observed in AD patients at Braak-Tau I/II stages, possibly before the appearance of the distinctive clinical symptoms of this condition. An aberrantly increased presence of cells with several primary apical dendrites is the first morphological change detected in DGCs in this disease. This alteration persists throughout AD progression and leads to generalized dendritic atrophy at advanced stages of the condition. Taken together, our data reveal the distinct vulnerability of several morphological features of inner and outer DGCs to AD and support the notion that the malfunction of the hippocampus is related to the cognitive impairments observed in AD patients.

## Supplementary Information

**Additional file 1: Figure S1.** Epidemiological data of the subjects included in this study. **a**: The subject code, clinical diagnosis, age, gender, post-mortem delay (PMD, i.e., the time lapse between exitus and tissue immersion in Golgi solution), Braak-Tau, and CERAD stages. **b** and **c**: Age of the subjects (**b**) and PMD of the samples (**c**) included in this study. n = 5 neurologically healthy control subjects and 17 AD patients.

**Additional file 2: Figure S2.** Delineation of inner and outer regions of the human granule cell layer (GCL). (**a**–**c**): Representative image of Golgi-stained human hippocampus and high-power magnification image showing the human GCL, and the method used to divide this layer into two inner and outer halves. Green bar: 500 µm. Yellow bar: 200 µm. Red bar: 20 µm.DG, dentate gyrus; GCL, granule cell layer; H, hilus; ML, molecular layer. Blue arrowhead: outer dentate granule cell (DGC). Red arrowhead: inner DGC.

**Additional file 3: Figure S3.** Correlations between morphometric determinations and the age of the subjects. **a**–**e**: Analyses of total DGCs. **a**: Correlation between total dendritic length and the age of the subjects. **b**: Correlation between the area of the soma and the age of the subjects. **c**: Correlation between the number of ending-tips and the age of the subjects. **d**: Correlation between the dendritic complexity index and the age of the subjects. **e**: Correlation between the maximum dendritic span and the age of the subjects. **f–j**: Analyses of outer DGCs. **f**: Correlation between total dendritic length and the age of the subjects. **g**: Correlation between the area of the soma and the age of the subjects. **h**: Correlation between the number of ending-tips and the age of the subjects. **i**: Correlation between the dendritic complexity index and the age of the subjects. **j**: Correlation between the maximum dendritic span and the age of the subjects. **k-o**: Analyses of inner DGCs. **k**: Correlation between total dendritic length and the age of the subjects. **l**: Correlation between the area of the soma and the age of the subjects. **m**: Correlation between the number of ending-tips and the age of the subjects. **n**: Correlation between the dendritic complexity index and the age of the subjects. **o**: Correlation between the maximum dendritic span and the age of the subjects.

**Additional file 4: Figure S4.** Morphological characteristics of human dentate granule cells (DGCs) located in the outer and inner granule cell layer (GCL) of neurologically healthy control subjects and patients with Alzheimer´s disease (AD). **a** Total dendritic length. **b** Area of the soma. **c** Number of ending-tips. **d** Percentage of cells with more than one apical primary dendrite. **e** Dendritic complexity index (DCI). **f** Maximum dendritic span. **g** Sholl´s analysis of outer DGCs. **h** Sholl´s analysis of inner DGCs. **i** Schematic representation of dendrite branch orders. **j**. Number of dendrites in each branch order of outer DGCs. **k** Number of dendrites in each branch order of inner DGCs. A colored asterisk indicates statistically significant changes with respect to control subjects. Black asterisk indicates changes between outer and inner DGCs. n = 127 cells obtained from 5 neurologically healthy control subjects and 512 cells obtained from 17 AD patients. * 0.05 > *P* ≥ 0.01; ** 0.01 > *P* ≥ 0.001; and *** *P* < 0.001. Asterisks represent statistically significant differences in Tukey`s (two-way ANOVA) or Dunn`s (Kruskal-Wallis) post-hoc analyses, or Chi-squared test.

**Additional file 5:** Extended Data: Detailed results from statistical comparisons. The results of all statistical comparisons included in this manuscript are presented. Each tab includes all the statistical comparisons that refer to a single Figure or Additional file.

## Data Availability

All the data generated and analyzed are included in the main or supplementary files of this manuscript.
