# Supplementary figures and images for: Progression of Alzheimer's disease parallels unusual structural plasticity of human dentate granule cells
Source: Acta Neuropathol Commun. 2022 Aug 29;10:125. doi: 10.1186/s40478-022-01431-7 (PMC9426249; doi:10.1186/s40478-022-01431-7)

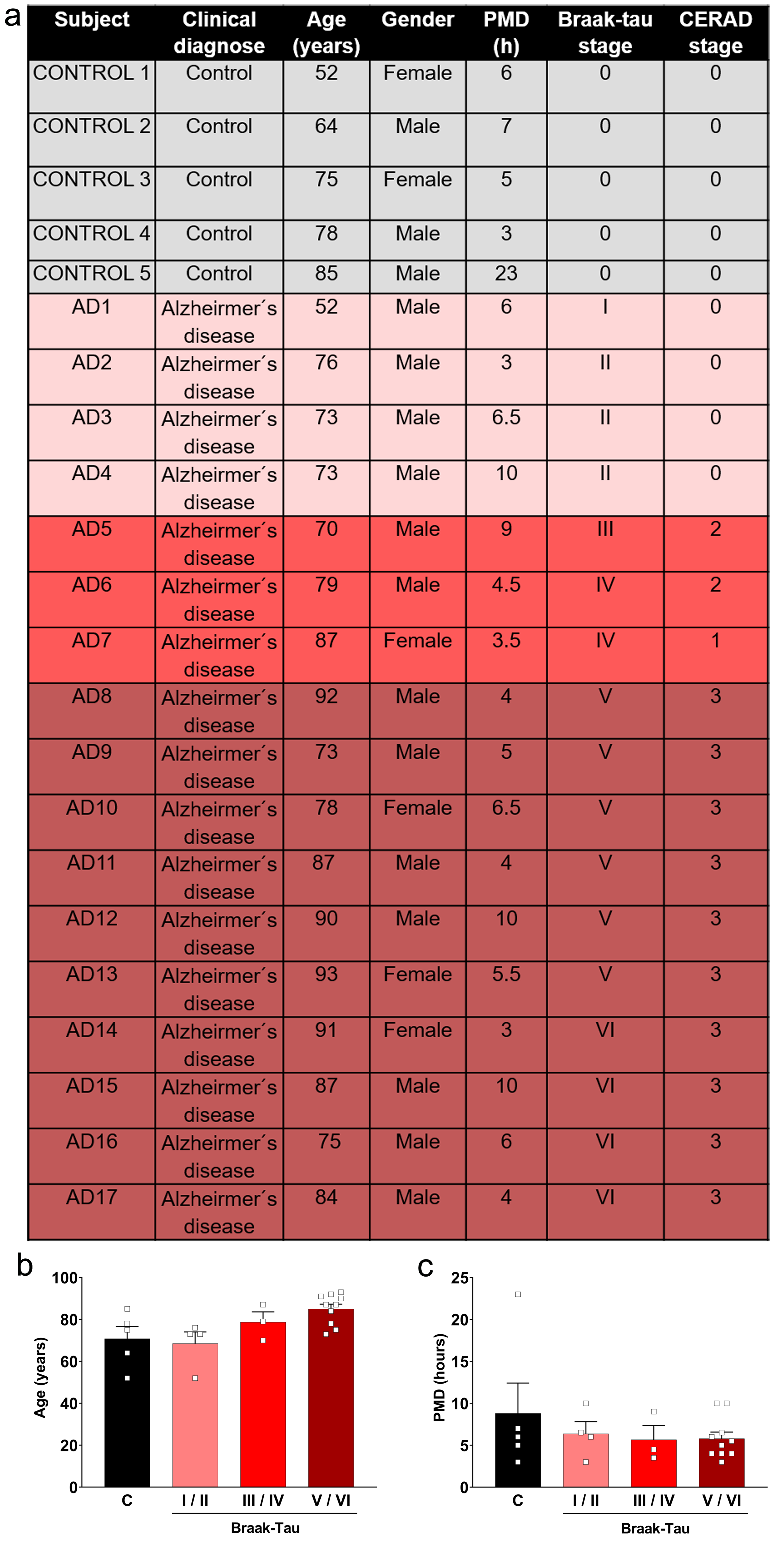

Supplement: Supplementary file 1 — Additional file 1: Figure S1. Epidemiological data of the subjects included in this study. a: The subject code, clinical diagnosis, age, gender, post-mortem delay (PMD, i.e., the time lapse between exitus and tissue immersion in Golgi solution), Braak-Tau, and CERAD stages. b and c: Age of the subjects (b) and PMD of the samples (c) included in this study. n = 5 neurologically healthy control subjects and 17 AD patients. [file 40478_2022_1431_MOESM1_ESM.tif]

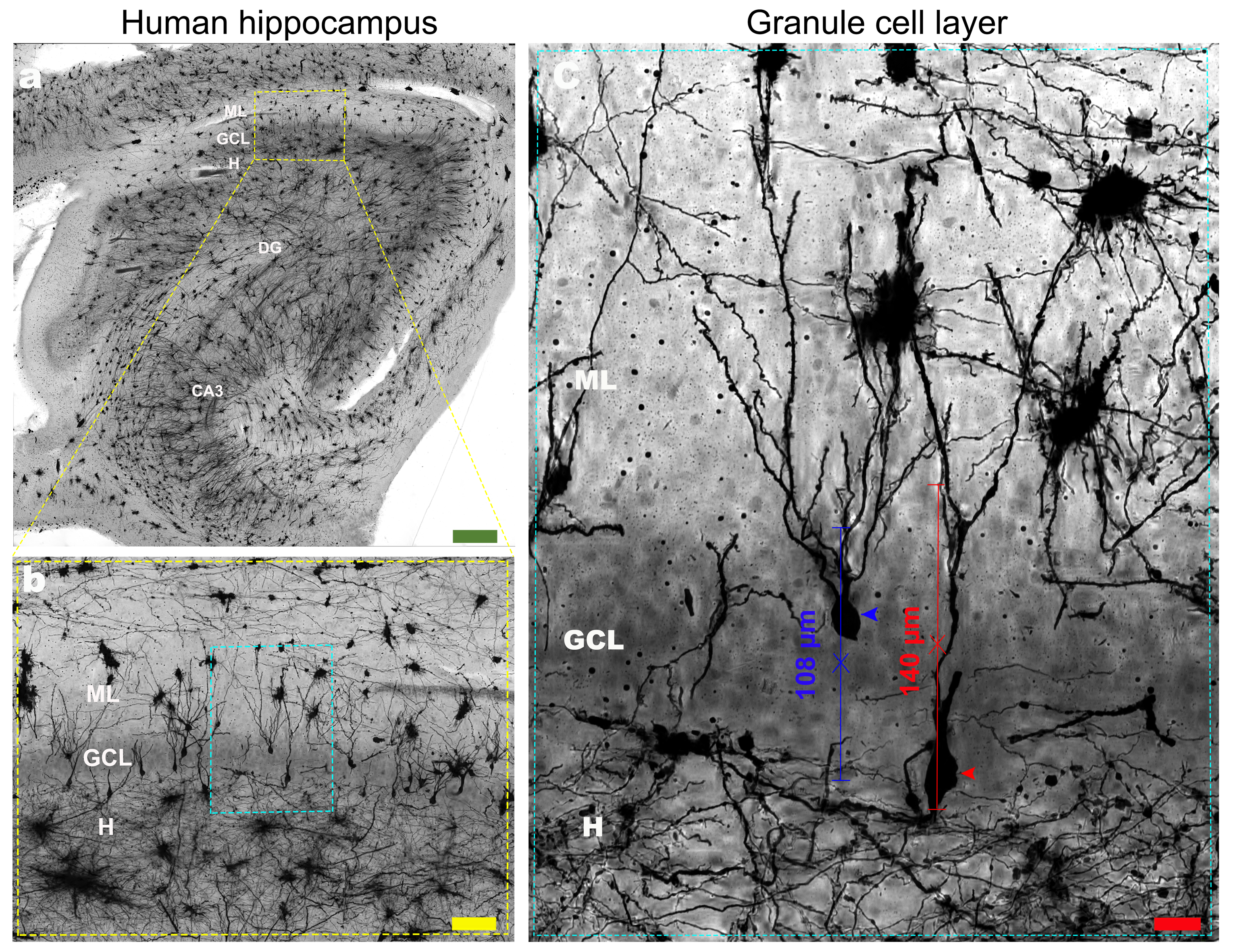

Supplement: Supplementary file 2 — Additional file 2: Figure S2. Delineation of inner and outer regions of the human granule cell layer (GCL). (a–c): Representative image of Golgi-stained human hippocampus and high-power magnification image showing the human GCL, and the method used to divide this layer into two inner and outer halves. Green bar: 500 µm. Yellow bar: 200 µm. Red bar: 20 µm.DG, dentate gyrus; GCL, granule cell layer; H, hilus; ML, molecular layer. Blue arrowhead: outer dentate granule cell (DGC). Red arrowhead: inner DGC. [file 40478_2022_1431_MOESM2_ESM.tif]

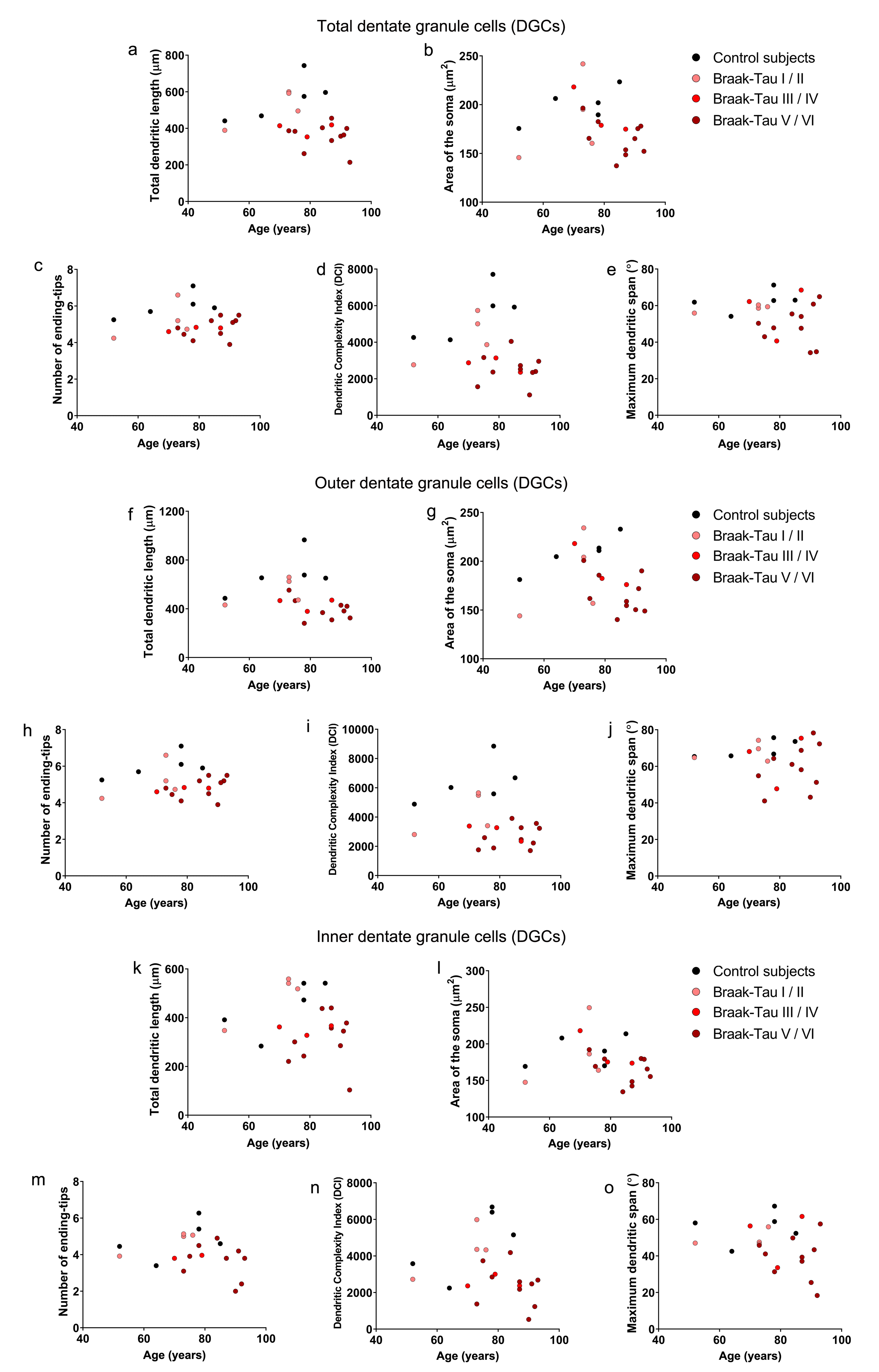

Supplement: Supplementary file 3 — Additional file 3: Figure S3. Correlations between morphometric determinations and the age of the subjects. a–e: Analyses of total DGCs. a: Correlation between total dendritic length and the age of the subjects. b: Correlation between the area of the soma and the age of the subjects. c: Correlation between the number of ending-tips and the age of the subjects. d: Correlation between the dendritic complexity index and the age of the subjects. e: Correlation between the maximum dendritic span and the age of the subjects. f–j: Analyses of outer DGCs. f: Correlation between total dendritic length and the age of the subjects. g: Correlation between the area of the soma and the age of the subjects. h: Correlation between the number of ending-tips and the age of the subjects. i: Correlation between the dendritic complexity index and the age of the subjects. j: Correlation between the maximum dendritic span and the age of the subjects. k-o: Analyses of inner DGCs. k: Correlation between total dendritic length and the age of the subjects. l: Correlation between the area of the soma and the age of the subjects. m: Correlation between the number of ending-tips and the age of the subjects. n: Correlation between the dendritic complexity index and the age of the subjects. o: Correlation between the maximum dendritic span and the age of the subjects. [file 40478_2022_1431_MOESM3_ESM.tif]

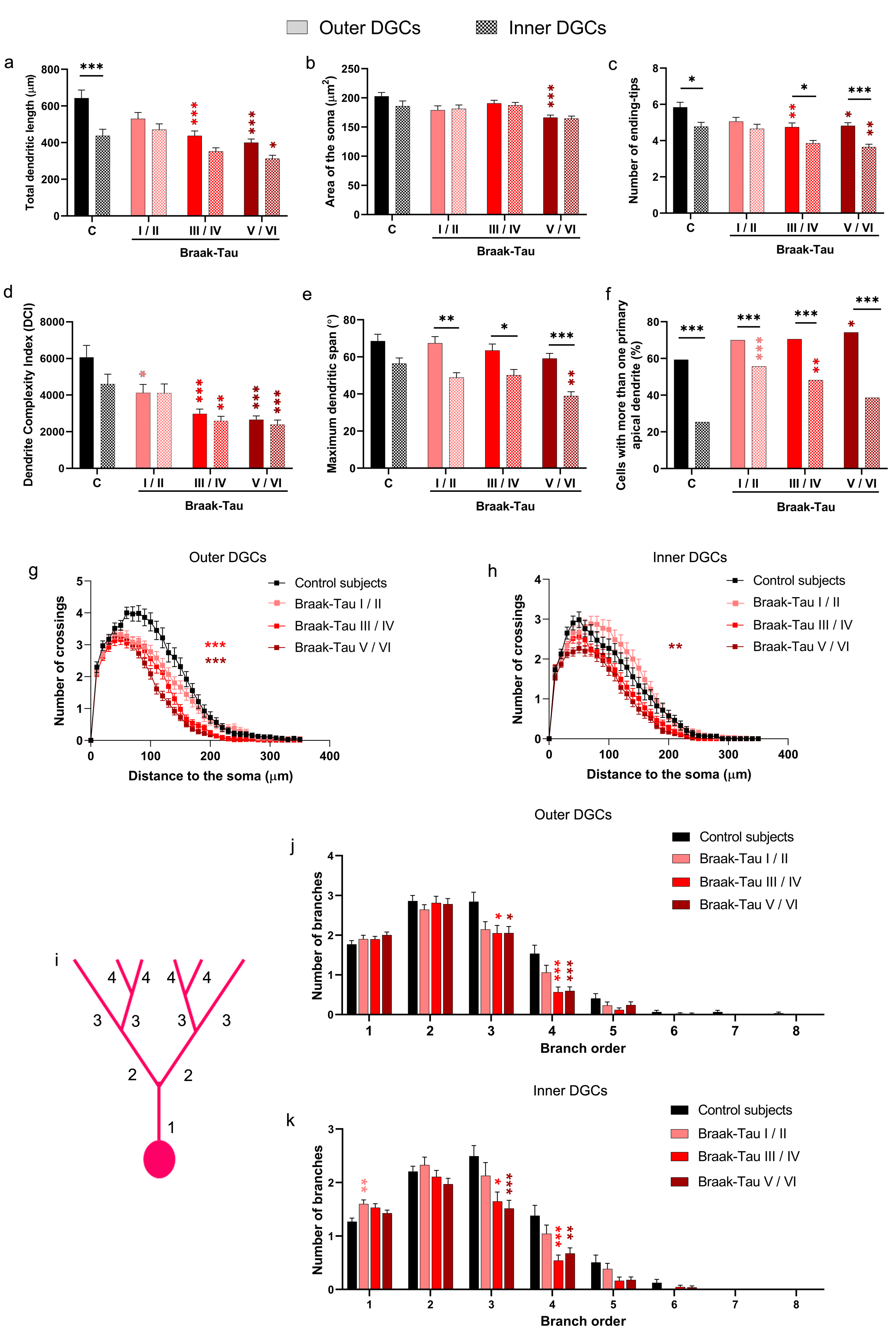

Supplement: Supplementary file 4 — Additional file 4: Figure S4. Morphological characteristics of human dentate granule cells (DGCs) located in the outer and inner granule cell layer (GCL) of neurologically healthy control subjects and patients with Alzheimer´s disease (AD). a Total dendritic length. b Area of the soma. c Number of ending-tips. d Percentage of cells with more than one apical primary dendrite. e Dendritic complexity index (DCI). f Maximum dendritic span. g Sholl´s analysis of outer DGCs. h Sholl´s analysis of inner DGCs. i Schematic representation of dendrite branch orders. j. Number of dendrites in each branch order of outer DGCs. k Number of dendrites in each branch order of inner DGCs. A colored asterisk indicates statistically significant changes with respect to control subjects. Black asterisk indicates changes between outer and inner DGCs. n = 127 cells obtained from 5 neurologically healthy control subjects and 512 cells obtained from 17 AD patients. * 0.05 > P ≥ 0.01; ** 0.01 > P ≥ 0.001; and *** P < 0.001. Asterisks represent statistically significant differences in Tukey`s (two-way ANOVA) or Dunn`s (Kruskal-Wallis) post-hoc analyses, or Chi-squared test. [file 40478_2022_1431_MOESM4_ESM.tif]
